# Distinct Immune Signatures Indicative of Treatment Response and Immune-Related Adverse Events in Melanoma Patients under Immune Checkpoint Inhibitor Therapy

**DOI:** 10.3390/ijms22158017

**Published:** 2021-07-27

**Authors:** Robin Reschke, Philipp Gussek, Andreas Boldt, Ulrich Sack, Ulrike Köhl, Florian Lordick, Thomas Gora, Markus Kreuz, Kristin Reiche, Jan-Christoph Simon, Mirjana Ziemer, Manfred Kunz

**Affiliations:** 1 Department of Dermatology, Venereology and Allergology, University Medical Center Leipzig, Philipp-Rosenthal-Str. 23, 04103 Leipzig, Germany; robin.reschke@medizin.uni-leipzig.de (R.R.); philipp.gussek@medizin.uni-leipzig.de (P.G.); thomas.gora@medizin.uni-leipzig.de (T.G.); Jan-Christoph.Simon@medizin.uni-leipzig.de (J.-C.S.); mirjana.ziemer@medizin.uni-leipzig.de (M.Z.); 2 Institute of Clinical Immunology, University Medical Center Leipzig, Johannisallee 30, 04103 Leipzig, Germany; andreas.boldt@medizin.uni-leipzig.de (A.B.); Ulrich.Sack@medizin.uni-leipzig.de (U.S.); ulrike.koehl@izi.fraunhofer.de (U.K.); Kristin.Reiche@izi.fraunhofer.de (K.R.); 3 Fraunhofer Institute for Cell Therapy and Immunology (IZI), Perlickstrasse 1, 04103 Leipzig, Germany; markus.kreuz@izi.fraunhofer.de; 4 Department of Oncology, Gastroenterology, Hepatology, Pulmonology and Infectious Diseases, University Medical Center Leipzig, Liebigstrasse 20, 04103 Leipzig, Germany; Florian.Lordick@medizin.uni-leipzig.de; 5 University Cancer Center Leipzig (UCCL), University Medical Center Leipzig, Liebigstrasse 22, 04103 Leipzig, Germany

**Keywords:** immunology, T cells, melanoma, flow cytometry, immune checkpoint

## Abstract

To identify potential early biomarkers of treatment response and immune-related adverse events (irAE), a pilot immune monitoring study was performed in stage IV melanoma patients by flow cytometric analysis of peripheral blood mononuclear cells (PBMC). Overall, 17 patients were treated with either nivolumab or pembrolizumab alone, or with a combination of nivolumab and ipilimumab every three weeks. Of 15 patients for which complete response assessment was available, treatment responders (*n* = 10) as compared to non-responders (*n* = 5) were characterized by enhanced PD-1 expression on CD8^+^ T cells immediately before treatment (median ± median absolute deviation/MAD 26.7 ± 10.4% vs. 17.2 ± 5.3%). Responders showed a higher T cell responsiveness after T cell receptor ex vivo stimulation as determined by measurement of programmed cell death 1 (PD-1) expression on CD3^+^ T cells before the second cycle of treatment. The percentage of CD8^+^ effector memory (CD8^+^CD45RA^−^CD45RO^+^CCR7^−^) T cells was higher in responders compared to non-responders before and immediately after the first cycle of treatment (median ± MAD 39.2 ± 7.3% vs. 30.5 ± 4.1% and 37.7 ± 4.6 vs. 24.0 ± 6.4). Immune-related adverse events (irAE) were accompanied by a higher percentage of activated CD4^+^ (CD4^+^CD38^+^HLADR^+^) T cells before the second treatment cycle (median ± MAD 14.9 ± 3.9% vs. 5.3 ± 0.4%). In summary, PBMC immune monitoring of immune-checkpoint inhibition (ICI) treatment in melanoma appears to be a promising approach to identify early markers of treatment response and irAEs.

## 1. Introduction

Cancer immunotherapies have a major impact on patient outcomes [1]. In particular, immunecheckpoint inhibition (ICI) strategies targeting the programmed cell death protein 1 (PD-1), programmed cell death protein ligand 1 (PD-L1), and cytotoxic T-lymphocyte-associated protein 4 (CTLA-4) are approved for a large number of different cancers [1]. The longest experiences with ICI therapies exist for melanoma, renal cell cancer, and non-small cell lung cancer. In melanoma, PD-1 and CTLA-4 inhibitory antibodies (nivolumab, pembrolizumab, and ipilimumab) have been approved as single agents or in combination for treatment of patients with unresectable stage III and distant metastatic disease [2,3]. Further, pembrolizumab, nivolumab, and ipilimumab have been approved by the U.S. Food and Drug Administration (FDA) for adjuvant melanoma therapy [4]. 

However, a significant number of patients still do not respond to these treatments (60% in case of PD-1 inhibition, 80% for CTLA4 inhibition). A variety of predictive biomarkers for treatment response have been proposed. A major predictor of clinical benefit to anti-PD-1 treatment is the presence of a T cell-inflamed gene expression profile in the tumor microenvironment [5,6]. In melanoma and head and neck cancer, baseline IFN-γ-related mRNA profiles were increased in tumors of responders to anti-PD-1 therapy [7]. In melanoma, the frequency of tumor antigen-specific CD8^+^ T cells residing in the tumor microenvironment found in baseline biopsies and during treatment was associated with therapeutic response [8,9]. Negative regulatory factors, such as PD-L1 on tumor cells, or the presence of FoxP3^+^ T regulatory cells (Tregs) and T cell anergy are observed in T cell-inflamed tumors and may act as markers for response prediction [10]. 

However, immune monitoring on metastasis tissues prior to treatment and during the course of therapy requires longitudinal biopsies, a rather impractical approach for most patients and clinical settings. Thus, the analysis of blood samples appears to be much more feasible and might even mirror the immunological environment in the tumor [11,12]. A study with the anti-PD1 antibody pembrolizumab using mass cytometry and flow cytometry of peripheral blood mononuclear cells showed that the reinvigoration of Ki67^+^ circulating exhausted-phenotype CD8^+^ T cells (T_ex_) in relation to pretreatment tumor burden correlated with clinical response [11]. However, response rates were also influenced to a certain degree by the amount of T cells (CD8^+^ and CD4^+^), myeloid cells, monocytes, and PD-1- and CTLA-4-expression on T-cells in the patients’ blood [11,13]. 

Only a few studies examined biomarkers predicting immune-related adverse events (irAEs) to ICI therapies. In a proteome analysis, specific pre-treatment IgG-antibody signatures in sera of melanoma patients predicted irAE development [14]. Furthermore, the expression of specific chemokines and interleukins (increases in CXCL9 and CXCL10) during therapy with PD-1 inhibitor could identify melanoma patients who are at high-risk for irAEs [15]. The analysis of T cell clonality from blood samples in patients with pancreatic cancer demonstrated that an increased amount of T cell clones could predict severe irAEs [16,17].

IrAEs are common in ICI therapy and may be explained by T cell reactivation. The anti-PD-1 antibodies nivolumab and pembrolizumab have a similar range of irAEs but are less frequent and, in the majority, less severe compared to ipilimumab. For patients treated with nivolumab, the most common irAEs were fatigue, rashes, diarrhea, pruritus, nausea, and endocrinopathies [18]. The most frequent and severe irAEs occur during combination therapy with nivolumab and ipilimumab. 

Taken together, current immunotherapies in melanoma are associated with stilllimited response rates and significant rates of immune-related adverse events. Thus, predictive clinical markers that might be obtained by easily accessible patient material such a blood samples, and also early during treatment, might help to improve clinical treatment decisions and patient monitoring. In the present study, a panel of more than 40 different immune cell subsets of peripheral blood mononuclear cells was analyzed in melanoma patients under ICI therapy. This exploratory study was performed to identify candidate cell subsets for future, more focused, studies of larger sample sets, which may ultimately be used in routine clinical settings to guide treatment decisions.

## 2. Results

### 2.1. General Clinical Characteristics

Seventeen patients (eight females, and nine males) were enrolled from June 2019 to October 2020. Apart from one mucosal melanoma and two melanomas of unknown primary, all patients had stage IV metastatic cutaneous melanoma. Ten patients were treatment-naïve. Eight patients started with the combination of ipilimumab and nivolumab, five patients were treated with pembrolizumab and four patients received nivolumab as a single agent. One patient who received the combination treatment was previously progressive in the course of adjuvant treatment with nivolumab and afterwards received targeted therapy with dabrafenib and trametinib and stereotactic radiation of a solitary brain metastasis. Another patient was pretreated in advanced stage IV with dabrafenib and trametinib and achieved complete remission but relapsed after a treatment-free interval of six months. The *BRAF* V600E mutation was observed in five patients. In ten patients, at least 2% of tumor cells expressed PD-L1. PD-L1 was negative in four patients (Table 1).

### 2.2. Immune-Related Adverse Events

Eight out of seventeen patients developed irAEs (47%) within the three months of immune monitoring (Table 1), four patients experienced multiple irAEs. IrAEs consisted of colitis, hepatitis, hypophysitis, nephritits, oral mucositis, pneumonitis, and thyroiditis. In 40% of these patients, the above mentioned irAEs were accompanied by fatigue, which varied between grade 1–3 (Common Terminology Criteria for Adverse Events, CTCAEv5.0). All patients who received the combination of ipilimumab and nivolumab experienced irAEs. Most of their irAEs were mild to moderate. One patient suffered from fatigue grade 3. Patient 7 had to stop ICI due to irAEs.

### 2.3. Tumor Response

Two patients achieved CR and eight patients PR (summarized as responders). One patient showed SD and four patients progressive disease (PD) (summarized as non-responders). Of these four, two patients died from disease progression. In two patients, response assessment was not available due to an early change of treatment regimen to targeted therapy (TT) (patient 12) and unknown cause of unexpected death in early treatment phase (patient 14) (Table 1). 

### 2.4. Immune Monitoring

The expression of checkpoint point molecules PD-1 and CTLA-4 as well as the percentages of different T cell, B cell and monocyte subpopulations at indicated time points were analyzed by flow cytometry (overall 87 immune features; Supplementary Table S2). Responder versus non-responder patients were compared as well as patients with and without irAEs. Only irAEs which occurred during the 3 months of the observational period were included into the study. Baseline expression values and experimentally induced expression of checkpoint molecules as well as irAEs-related values were of particular interest. 

**Figure 1:**
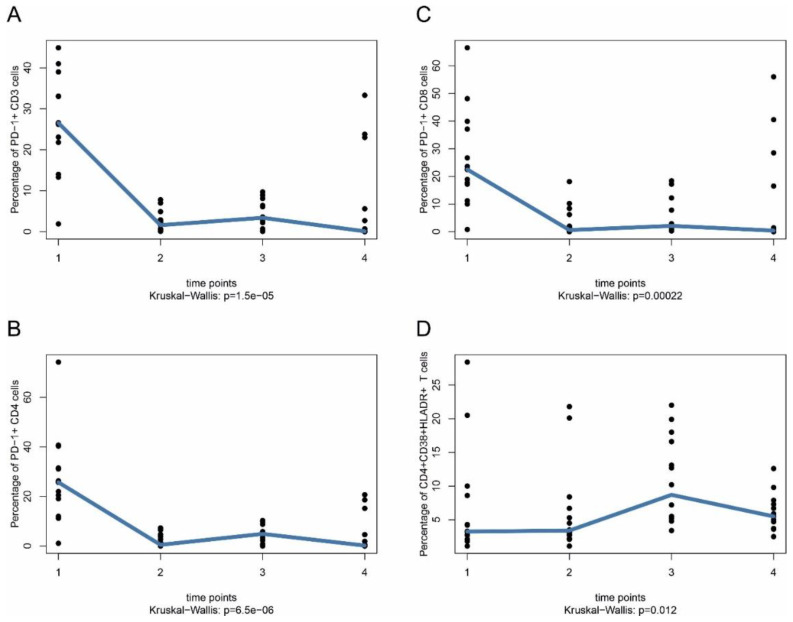
PD-1 expression on T cells in stage IV melanoma patients under ICI therapy. PBMC were taken from patients at indicated time points and PD-1 expression on T cells was analyzed by flow cytometry (FACS). Blood samples were taken immediately before first treatment with anti-PD-1 (either alone or in combination with anti-CTLA-4) (time point 1), immediately after first treatment on the same day (time point 2), after two or three weeks (time point 3) and after three months (time point 4). (**A**) PD1^+^CD3^+^ T cells. (**B**) PD1^+^CD4^+^ T cells. (**C**) PD1^+^CD8^+^ T cells. (**D**) CD4^+^CD38^+^HLADR^+^ T cells. Nominal (unadjusted) *p*-values are shown without adjustment for multiple testing. Data in (**A**–**C**) are given as percentage of PD-1^+^ cells of total CD3^+^, CD4^+^, and CD8^+^ T cells, respectively. Data in (**D**) are given as percentage of CD38^+^HLADR^+^ cells of total CD4^+^ T cells.

The strongest time course effects were seen for PD-1 downregulation on CD3^+^, CD4^+^, and CD8^+^ T cells at all time points after start of treatment (CD3^+^: median 26.6% vs. medians 0.1–3.4% for other time points; CD4^+^: median 25.7% vs. medians 0.2–4.9% for other time points; CD8: median 22.5% vs. medians 0.4–2.1% for other time points), which is most likely due to the PD-1 blocking antibodies used for treatment (Figure 1A–C). Time course analysis also showed that the median of the percentages of activated (CD4^+^CD38^+^HLA-DR^+^) CD4^+^ T cells (*p* = 0.012) increased over time, starting with a median of 3.3% (time point 1) vs. medians 3.4% (time point 2), 8.7% (time point 3) and 5.5% (time point 4) (Figure 1D).

**Figure 2:**
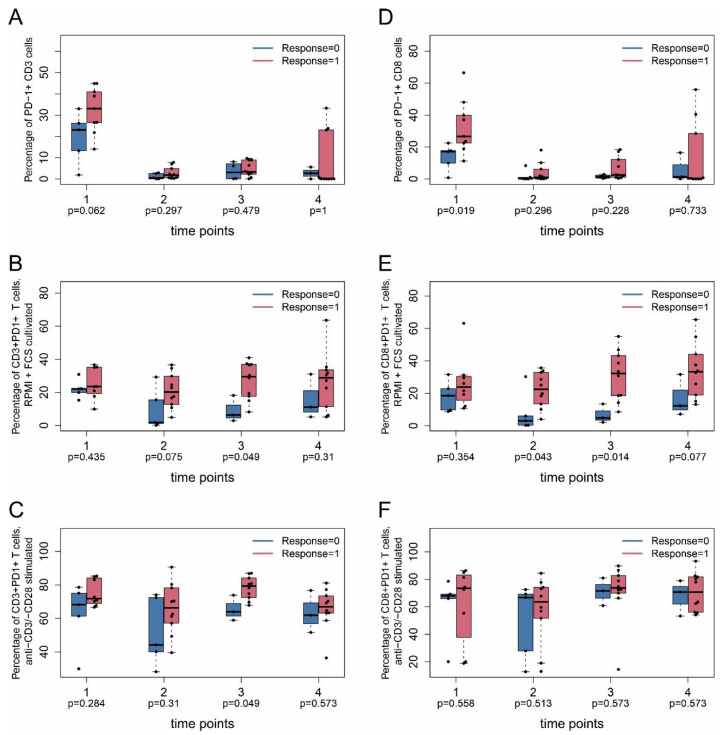
PD-1 expression on CD3^+^ and CD8^+^ T cells in ICI responders and non-responders. PBMC were taken from patients at indicated time points as described in Figure 1 and analyzed by flow cytometry (FACS). (**A**,**D**) PD1^+^CD3^+^ or PD1^+^CD8^+^ expression without stimulation. (**B**,**E**) PD1^+^CD3^+^ or PD1^+^CD8^+^ expression in control cells after cell culture in 10% FCS. (**C**,**F**) PD1^+^CD3^+^ or PD1^+^CD8^+^ expression after anti-CD3/anti-CD28 stimulation. Nominal (unadjusted) *p*-values (Mann–Whitney U test between responders and non-responders) are shown without adjustment for multiple testing. Responders are indicated by response = 1, non-responders by response = 0. Data in (**A**–**F**) are given as percentage of PD-1^+^ cells of total CD4^+^ and CD8^+^ T cells, respectively.

A number of markers were differentially expressed between responders and non-responders (Figure 2A–F). There was a consistent trend for PD-1 expression on CD3^+^ T cells in responders compared to non-responders without stimulation (median ± MAD 33.1 ± 7.9% vs. 23.1 ± 9.8%) (Figure 2A). Latent T cell responsiveness to external stimuli and to local stimuli present in the tumor microenvironment might be factors that influence treatment response. To address this issue, PBMC were washed and subsequently stimulated for 24 h with anti-CD3/anti-CD28 at every time point of treatment to activate T cell receptor signaling. Control cells were kept in T cell standard culture medium containing 10% FCS. PD-1 surface expression was measured as a surrogate marker for T cell re-activation. Control T cells kept under serum-conditions as well as anti-CD3/anti-CD28-stimulated T cells showed more prominent PD-1 expression on CD3^+^ T cells in responders compared to non-responders at time point 3 (median ± MAD 29.6 ± 9.0% vs. 6.3 ± 3.4% and 79.3 ± 5.8% vs. 63.9 ± 5.0%) (Figure 2B,C). This difference was not observed for CD4^+^ T cells (Supplementary Figure S1).

PD-1 expression on CD8^+^ T cells was significantly higher at time point 1 (immediately before first treatment) in responders than in non-responders (median ± MAD 26.7 ± 10.4% vs. 17.2 ± 5.3%) (Figure 2D), but this difference was lost at later time points. Control T cells kept under serum-conditions as well as anti-CD3/anti-CD28-stimulated T cells showed more prominent PD-1 expression on CD8^+^ T cells under serum-conditions at time points 2 and 3 (median ± MAD 22.5 ± 9.8% vs. 3.1 ± 2.8% and 32.1 ± 13.4% vs. 4.9 ± 2.7%) (Figure 2E). The difference was no longer present after stimulation of CD8^+^ T cells with anti-CD3/anti-CD28 antibodies (Figure 2F). Here, stimulation with anti-CD3/anti-CD28 induced close to 80% positive cells in both responders and non-responders, and differences observed for cells kept under serum-conditions may be leveled out under anti-CD3/anti-CD28 stimulatory conditions (Figure 2F). Taken together, latent T cell responsiveness of CD8^+^ T cells might be a valuable early marker for treatment response. 

**Figure 3:**
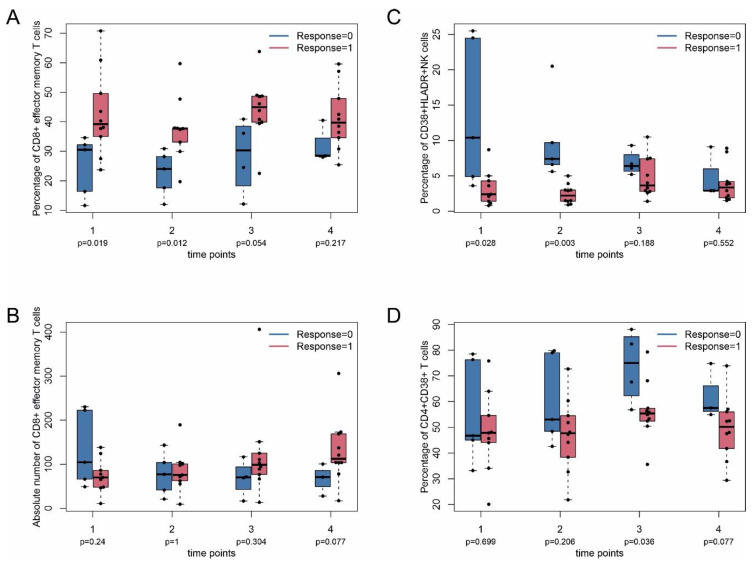
Inflammatory T cell subpopulations in ICI responders and non-responders. PBMC were taken from patients at indicated time points as described in Figure 1 and analyzed by flow cytometry (FACS). (**A**,**B**) Percentage and absolute number of effector memory T cells, respectively. (**C**) CD38^+^HLADR^+^ NK cells. (**D**) CD4^+^CD38^+^ cells. Nominal (unadjusted) *p*-values (Mann–Whitney U test between responders and non-responders) are shown without adjustment for multiple testing. Responders are indicated by response = 1, non-responders by response = 0. Data in (**A**) are given as percentage of CD8^+^ effector memory cells of total CD8^+^ T cells. Absolute number refers to CD8^+^ effector memory cells per μL. Data in (**C**) are given as CD38^+^HLADR^+^ cells of total NK cells. Data in (**D**) are given as percentage of CD38^+^ cells of total CD4^+^ cells.

Responders also had significantly higher percentage of CD8^+^ (CD8^+^CD45RA^−^CD45RO^+^CCR7^−^) T effector memory T cells than non-responders at time points 1 and 2 (median ± MAD 39.2 ± 7.3% vs. 30.5 ± 4.1% and 37.7 ± 4.6 vs. 24.0 ± 6.4) (Figure 3A). The absolute number of CD8+ effector memory T cells was not different between responders and non-responders.

The percentage of activated (CD16^+^CD56^+^CD38^+^HLA-DR^+^) NK cells was higher in non-responders at time points 1 and 2 (median ± MAD 10.4 ± 6.8% vs. 2.4 ± 1.3% and 7.4 ± 1.8% vs. 2.2 ± 0.8%) (Figure 3C). The percentage of activated CD4^+^ T cells (CD4^+^CD38^+^) was significantly higher at time point 3 in non-responders compared with responders (median ± MAD 75.0 ± 10.2% vs. 55.3 ± 2.5%) (Figure 3D). In line with this, the percentage of activated CD4^+^ T cells (CD4^+^CD38^+^HLA-DR^+^) increased over time, as described above (Figure 1D). Taken together, CD8^+^ effector memory T cells, activated (CD16^+^CD56^+^CD38^+^HLA-DR^+^) NK cells, and CD4^+^CD38^+^ T cells may be early markers of treatment response. pSTAT5 at position 705 (pY705) is regarded as a general marker for T cell activation [19]. The baseline value of pSTAT5 in T cells at time point 1 was significantly higher in responders (Supplementary Figure S2). However, the baseline values of all patients were very low (below 5%). The clinical relevance of this finding is unclear at the moment and requires further investigation. No difference was observed after T cell stimulation with IL-2 or anti-CD3/anti-CD28 for pSTAT5, which, however, dramatically increased pSTAT5 values (Supplementary Figure S2). 

**Figure 4:**
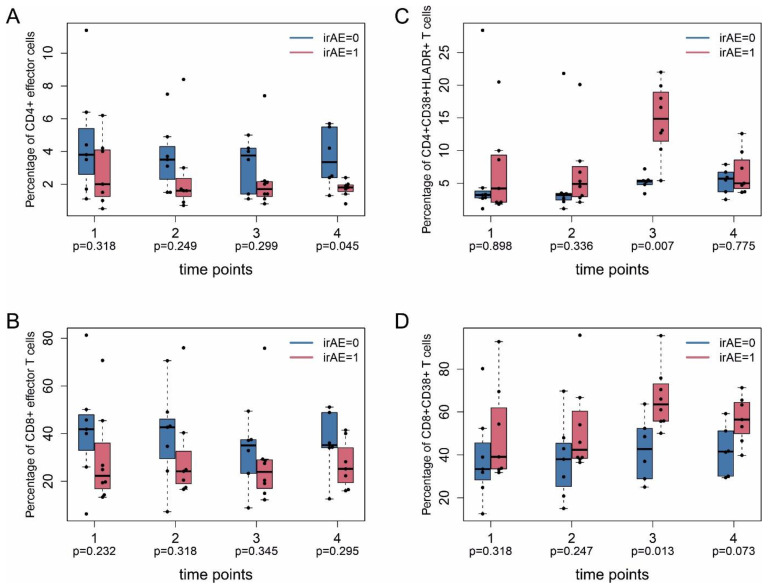
Inflammatory T cell subpopulations in patients with and without irAE. PBMC were taken from patients at indicated time points as described in Figure 1 and analyzed by flow cytometry (FACS). (**A**,**B**) Percentage of CD4^+^ and CD8^+^ effector T cells. (**C**) CD4^+^CD38^+^HLADR^+^ T cells. (**D**) CD8^+^CD38^+^ T cells. irAE, immune-related adverse events. 0, without; 1, with adverse events. Nominal (unadjusted) *p*-values (Mann–Whitney U test between irAE = 1 and irAE = 0) are shown without adjustment for multiple testing. Data in (**A**,**B**) are given as percentage of CD4^+^ effector cells and CD8^+^ effector cells, respectively, of total CD4^+^ and CD8^+^ cells. Data in (**C**) are given as percentage of CD38^+^HLADR^+^ cells of total CD4^+^ cells. Data in (**D**) are given as percentage of CD38^+^ cells of total CD8^+^ cells.

The proportion of effector T cells distinguished patients with irAEs from patients without irAEs. The percentage of CD4^+^ effector T cells (CD4^+^CD45RA^+^CD45RO^−^CCR7^−^) was lower at time point 4 (median ± MAD 2.0 ± 1.5% vs. 3.8 ± 2.1%) for patients with irAEs (Figure 4A). The percentage of CD8^+^ (CD8^+^CD45RA^+^CD45RO^−^CCR7^−^) effector T cells showed no difference in patients with irAEs (Figure 4B). The percentage of CD4^+^CD38^+^HLA-DR^+^ (Figure 4C) T cells at time point 3 was higher in patients with irAEs (median ± MAD 14.9 ± 3.9% vs. 5.3 ± 0.4%), as was the percentage of CD4^+^HLADR^+^ T cells (median ± MAD 24.8 ± 3.0% vs. 10.4 ± 1.8%) (Figure 4D), similar to the percentage of CD8^+^CD38^+^ T cells at time point 3 (median ± MAD 63.5 ± 7.8% vs. 42.8 ± 11.7%) (Figure 4D).

**Figure 5:**
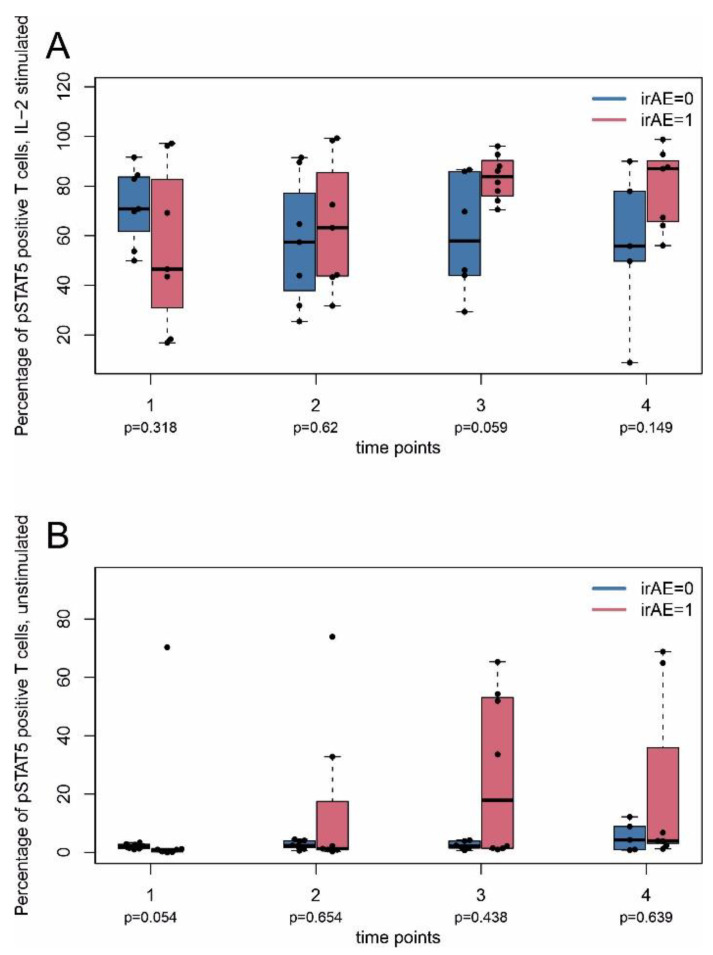
Intracellular expression of phosphorylated STAT5 (pSTAT5) in T cells. PBMC were taken from patients at indicated time points as described in Figure 1 and analyzed by flow cytometry (FACS). (**A**) pSTAT5 expression after T cell stimulation with IL-2 for 15 min. (**B**) pSTAT5 expression without T cell stimulation. irAE, immune-related adverse events. 0, without; 1, with adverse events. Nominal (unadjusted) *p*-values (Mann–Whitney U test between irAE = 1 and irAE = 0) are shown without adjustment for multiple testing. Data in (**A**,**B**) are given as percentage of pSTAT5 positive cells of total CD3^+^ T cells.

Baseline expression (time point 1) of phosphorylated STAT5 at position 705 (pY705) and stimulation with IL-2 showed that patients with irAE had a consistent trend of difference of pSTAT5 levels compared with patients without irAE at time point 3, possibly arguing for an enhanced responsiveness of lymphocytes of these patients (median ± MAD 83.8 ± 7.4% vs. 57.9 ± 21.0%) (Figure 5A,B). 

**Figure 6:**
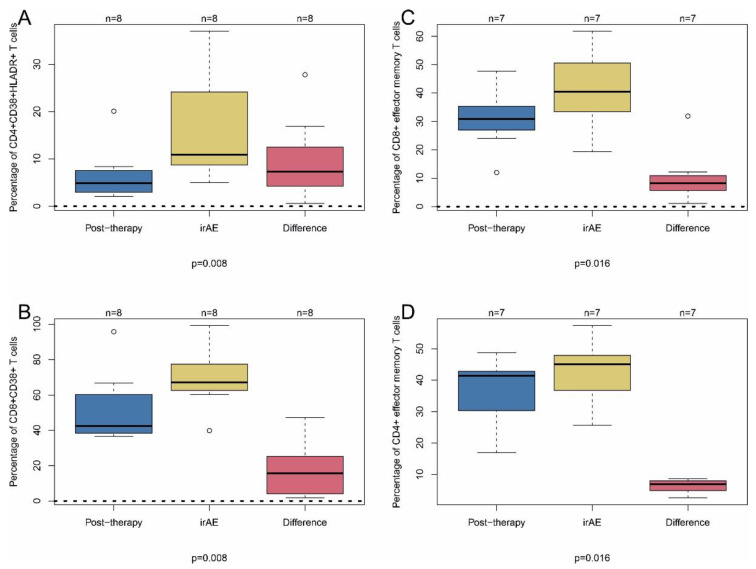
Inflammatory T cell subpopulations in patients with irAE. PBMC were taken from patients immediately after first treatment on the same day (post-treatment; time point 2 in previous figures). In addition, PBMC were taken from patients at the time point of first appearance of adverse events (irAE) and analyzed by flow cytometry (FACS). The red boxplots illustrate the pairwise difference of post-first treatment and irAE measurements. (**A**) CD4^+^CD38^+^HLADR^+^ T cells. (**B**) CD8^+^CD38^+^ T cells. (**C**) CD8^+^ effector memory T cells. (**D**) CD4^+^ effector memory T cells. Nominal (unadjusted) *p*-values (Wilcoxon signed-rank test between post-therapy and irAE) are shown without adjustment for multiple testing. Data in (**A**) are given as percentage of CD38^+^HLADR^+^ cells total CD4^+^ cells. Data in (**B**) are given as percentage of CD38^+^ cells of total CD8^+^ cells. Data in (**C**,**D**) are given as percentage of CD4^+^ and CD8^+^ effector memory cells, respectively, of total CD4^+^ and CD8^+^ cells.

Next, we analyzed markers of leukocyte activation at the time point of the occurrence of irAE (earliest time point after irAE and admission to our outpatient clinic with ongoing adverse events). We compared these values with time point 2 (immediately after the first intravenous administration of the respective immune therapy to exclude treatment-related PD-1 downregulation as a confounding parameter). In line with the time course of T cell activation, there was an induction of CD4^+^CD38^+^HLA-DR^+^ T cells (increase of 7.3 ± 3.1%), and of CD8^+^CD38^+^ T cells (increase of 15.6 ± 11.6%) at the time point of adverse events (Figure 6A,B). 

Furthermore, the percentage of CD8^+^ and CD4^+^ effector memory T cells (CD8^+^/CD4^+^CD45RA^−^CD45RO^+^CCR7^−^) was higher at this time point (increase of 8.2 ± 4.0% and 6.9 ± 1.8%, respectively) (Figure 6C,D). Moreover, the percentage and the absolute number of B cells was downregulated at this time point (Supplementary Figure S3). Thus, activated CD4^+^ and CD8^+^ T cells and CD4^+^/CD8^+^ effector memory T cells may indicate tumor response and adverse events. 

## 3. Discussion 

In an attempt to identify predictive immune markers for ICI treatment response and irAE, this exploratory study analyzed sequential blood samples in 15 stage IV melanoma patients under ICI therapy by flow cytometry for a comprehensive set of immune phenotypes. 

The strongest effects were seen for loss of PD-1 detection on CD3^+^, CD4^+^ and CD8^+^ T cells. Of note, this effect was seen immediately after the first ICI treatment, possibly due to the use of PD-1-blocking antibodies given either alone or in combination with anti-CTLA-4 treatment. PD-1 expression also characterized immune phenotypes of treatment responders and non-responders. The proportion of PD-1^+^CD8^+^ T cells in responders was significantly higher at time point 1 (before treatment) in responders compared to non-responders. This difference was lost during the further course treatment, likely due to PD-1 saturation by the applied antibodies. Our observations are in line with a study in non-small cell lung cancer patients treated with PD-1 inhibitor therapy, in which responding patients showed higher numbers of PD-1^+^CD8^+^-expressing T cells during the first four weeks of treatment, supporting a predictive role of PD-1 expression for ICI treatment [20].

In the present study, PD-1 expression on T cells remained low throughout three months of monitoring. However, PD-1 re-expression could be achieved by anti-CD3/-CD28 stimulation of lymphocytes ex vivo or under culture conditions of lymphocytes with FCS. In case of anti-CD3/-CD28 treatment, PD-1 expression was induced in ~80% of CD3^+^ and CD8^+^ lymphocytes. In case of CD3^+^ T cells, treatment responders showed a stronger responsiveness to anti-CD3/-CD28 stimulation (~82% positive cells), as compared to non-responders (~64% positive cells) at time point 3 (2 or 3 weeks after onset of treatment). These findings may thus help to identify non-responders at a relatively early time point. This difference was also observed in CD3^+^ and CD8^+^ lymphocytes kept in RPMI with FCS, which may represent a kind of serum stimulation. In line with these observations, CD28 expression (and activation) was shown to be important for the rescue of exhausted T cells under PD-1 targeted treatment [21]. The role of T cell receptor (TCR) activation for PD-1 induction, as observed in our study, is not completely understood, but PD-1 induction after TCR stimulation either alone or in combination with immune-activating cytokines has been reported in a number of earlier studies [22,23]. Thus, latent responsiveness to TCR stimulation appears to be a promising functional marker for treatment response in different cancer settings, which, to the best of our knowledge, has not been described in detail so far. In support of these findings, local PD-1 expression in melanoma tissues after stimulation in the local tissue microenvironment has been shown to have a prognostic value in ICI treatment settings [8]. Moreover, in an earlier report, T cell recovery of tumor-infiltrating lymphocytes (TIL) extracted from NSCLC biopsies could be observed within hours ex vivo in cell culture [24].

A very recent report of melanoma treatment-related immune monitoring showed that PD-1 downregulation on CD4^+^CD25^+^CD127^−^PD1^+^ regulatory T lymphocytes under PD-1 inhibitor treatment was observed only in patients with favorable prognosis but not in patients with unfavorable prognosis [25]. In this latter study, no reduction of PD-1 expression was observed in non-responders. However, based on a current review on different PD-1 staining antibodies, so far no reliable staining was described for any of the commonly used FACS antibodies after PD-1 inhibitor therapy [26]. Thus, the constant staining for PD-1 in unfavorable prognosis patients in this study cannot be explained at the moment. Regulatory T cells as a whole did not show a significant difference between populations with favorable or unfavorable prognosis in this study [25], which is supported by our data. 

In line with our observation of latent and enhanced responsiveness, proliferation marker Ki67 was highly expressed in PD-1^+^CD8^+^ T cells in another melanoma study after three weeks of treatment with the anti-PD1-inhibitor, an analysis performed with PMBC of 29 stage IV melanoma patients [11]. Moreover, a higher ratio of PD-1^+^Ki67^+^CD8^+^ (re-invigorated) CD8^+^ T cells to tumor burden was associated with better clinical response. T cell re-invigoration might indeed be a marker of enhanced responsiveness. The authors regarded CD38 and HLA-DR, the expression of which increased under treatment on CD4^+^ T cells in the present study, as cell surface surrogate markers for Ki67^+^ cells. 

Responders also had a higher percentage of CD8^+^ (CD8^+^CD45RA^−^CD45RO^+^CCR7^−^) effector memory T cells than non-responders in the present study. Thus, CD8^+^ effector memory T cells might be a second early marker for treatment response, since differences were already observed before the second cycle of treatment. Frequencies of CD4^+^ effector memory CD4^+^CD45RO^+^CD62L^−^ T cells were lower before therapy for responders in a recent study using cytometry by time of flight (CyTOF) for PBMC analysis [13]. This study analyzed 20 stage IV melanoma patients treated with anti-PD-1 antibodies. However, the CD8^+^ T cell subpopulation of responders had a higher frequency of central memory (CD8^+^CD45RO^+^CD62L^+^) T cells before and after 12 weeks of treatment than non-responders. High baseline CD14^+^CD16b^−^HLA-DRhi monocytes in PBMC were the most prominent predictor for progression-free and overall survival in this latter study [13]. The percentage of activated HLA-DR-positive monocytes was slightly higher in pretreatment samples in our study, but values did not reach statistical significance.

Further, effector memory T cells have been shown to be associated with durable responses in ICI-treated melanoma patients in a very recent study [27]. In this latter study, a large transcriptomic analysis of peripheral blood CD8^+^ lymphocytes was performed for metastatic melanoma patients receiving anti-PD-1 or anti-CTLA-4 therapy. The number of large TCR clones, as determined by TCR sequencing, was higher in responders compared to non-responders, which correlated with the percentage of CD8^+^, but not CD4^+^, effector memory T cells in peripheral blood. 

Moreover, transcriptomic and immune profiling were performed on 158 tumor biopsies from melanoma patients treated with anti-PD-1 monotherapy or combined anti-PD-1 and anti-CTLA-4 therapy. As determined by mass cytometry of melanoma tissues using a panel of 43 markers, CD8^+^/CD4^+^EOMES^+^CD69^+^CD45RO^+^ effector memory T cells were significantly more abundant in responders of combined immunotherapy compared with non-responders [28].

In a further study, thousands of immune cells from 48 tumor samples of melanoma patients were analyzed using single-cell gene expression profiles of lesional immune cells from metastatic melanomas under ICI (predominantly anti-PD-1) treatment [29]. In line with our findings, clusters of CD4^+^ and CD8^+^ effector memory cells were associated with response to treatment, and non-responder T cells expressed high levels CD38, LAG3 and HAVCR2 (TIM-3), well-known T cell exhaustion markers [29]. 

In line with this, one major finding in our study was that activated HLA-DR^+^CD38^+^NK cells and CD4^+^CD38^+^ T cells showed higher levels in non-responders as compared to responders. Similar findings have been reported in an experimental murine melanoma study that also included a number of patient samples [12]. Authors found that priming of T cells by a gp100 peptide vaccination strategy in parallel to anti-PD-1 treatment reversed the inhibitory effects of PD-1^+^CD38highCD8^+^ T cells as did the depletion PD-1^+^CD38highCD8^+^ cells. These findings emphasized the immune inhibitory role of CD38^+^ T cells. In patient samples, the fraction of PD1^+^CD38^+^CD8^+^ T cells was higher in cells extracted from melanoma lesions in non-responders compared to responders [12]. The CD38^+^ fraction in PD-1^+^CD8^+^ cells showed a similar behavior in PBMC. Thus, apart from PD-1, CD38 appears to be a significant inhibitor molecule for immune activation. The role of CD38 is incompletely understood in the immune context but its effects might be mediated by the metabolic CD38-NAD^+^ axis [30]. T cells with reduced surface expression of CD38 exhibited higher NAD^+^, mediating oxidative phosphorylation, higher glutaminolysis, and mitochondrial dynamics. Consequently, CD38 has gained interest as new molecule for targeted treatment of cancer as recently shown for multiple myeloma [31]. 

A major question of the present study was the role of immune phenotypes as predictors for the occurrence of irAE. In line with the time course of T cell activation, there was a prominent induction of CD4^+^CD38^+^HLA-DR^+^ T cells and of CD8^+^CD38^+^ T cells at the time point of adverse events, both subpopulations of activated CD4^+^ and CD8^+^ T cells. As mentioned above, the role of CD38 is not completely understood in tumor immunology, but overexpression might indicate suppressive mechanisms to control an over-activated immune system. Furthermore, the percentage of CD4^+^ and CD8^+^ (CD4^+^/CD8^+^CD45RA^−^CD45RO^+^CCR7^−^) effector memory T cells was higher at this time point. Thus, CD4^+^ and CD8^+^ effector memory T cells appear to indicate both tumor response and adverse events. Since this CD8^+^ T cell population was already present at time points 1 and 2 (before and immediately after the first treatment) in the responder versus non-responder analysis, it may serve as an early marker for response and irAE. Evidence has been provided that irAEs correlate with treatment response in ICI therapy using nivolumab [32]. Thus, CD4^+^/CD8^+^ effector memory T cells might be a link between both treatment response and irAE. Similar results were obtained in a very recent report, where it was demonstrated that a subset of patients predisposed to ICI-related hepatitis may be identified by expanded CD4^+^ effector memory T cells [33]. 

Overall, the number of studies analyzing markers of irAE in peripheral blood is limited, and so far, we have provided one of the most comprehensive studies. In the present study, we took blood samples from patients at up to five different time points within the first three months of therapy, where irAEs are most prevalent [34]. Future investigations are needed and should analyze the importance of the overall absolute number versus the percentage of the respective immune cell subpopulation, respectively.

Before the second treatment cycle (time point 3), the frequency of activated CD4^+^ T cells (CD4^+^CD38^+^HLA-DR^+^) was elevated and preceded the onset of irAEs. Similarly, the percentage of pSTAT5 expressing T cells (after 15 min of IL-2-stimulation) was higher in irAE patients. Thus, latent responsiveness of peripheral blood cells might also be a marker for irAE, as described above for treatment response. Activated (phosphorylated) STAT5 is a well-known marker for T cell proliferation and activation [19,35,36]. In accordance with these findings, the expression of proliferation markers on CD4^+^ T cells 2 weeks after therapy initiation of ipilimumab and GM-CSF was increased in patients with metastatic prostate cancer [17]. In this study, a diversification in the T cell repertoire of both CD4^+^ and CD8^+^ T cells was associated with the occurrence of irAEs, which was, however, shown in only two patients. Similarly, an increased number of activated Ki67^+^CD8^+^ T cells was associated with the development of irAEs in melanoma patients, 6 months after therapy initiation with adjuvant ipilimumab [24].

In line with our data of activated (CD4^+^CD38^+^HLADR^+^) T cells associated with irAE, Subudhi and coworkers showed that expansion of specific CD8^+^ Tcell clones preceded the development of severe irAE and identified CD8^+^ T cells as a potential predictive biomarker for irAEs in patients with metastatic prostate cancer under ipilimumab therapy [16]. Histopathological examinations of immune-related hepatitis in melanoma patients under either nivolumab or ipilimumab demonstrated a primarily CD8^+^ T lymphocytic infiltration of hepatic tissue [37]. Similarly, histopathologic examination of cardiac tissue from a patient who suffered from immune-related myocarditis under pembrolizumab showed a predominantly CD8^+^ T cell infiltration [38]. An earlier study showed that a low percentage of Ki67^+^EOMES^+^CD4^+^ T cells at baseline (but not under treatment) was associated with the occurrence of irAE in a study using adjuvant treatment of stage III and stage IV melanoma patients with anti-CTLA antibody ipilimumab [39]. This is partly in contrast to our study, as we did not observe differences in cell populations at baseline. However, the majority (59%) of the patients in the mentioned study received prior immunotherapy with a cell vaccine, GM-CSF, high-dose IL-2, or interferon-α, or biochemotherapy. Thus, a significant number of their baseline values might have been influenced by prior immune treatment, and thus these data might not be directly comparable to ours. 

The percentage and absolute numbers of B cells was reduced at the time point of irAE. The role of B cells in tumor immunology is still controversial at the moment and is related to positive and negative prognoses, respectively. However, evidence has been provided that a decline in B cell numbers is associated with irAE under ICI treatment of melanoma patients, in line with the results in the present study [40]. 

The present study has limitations such as the limited number of patients as it was designed as an exploratory study to find candidates for markers that may be validated in larger studies in future. However, the number of parameters analyzed exceeds that of many other studies, with additional ex vivo stimulation experiments. Moreover, time course analyses performed herein may add additional information about the validity of individual markers.

Taken together, the present comprehensive study of peripheral blood markers for treatment response and irAE is consistent with a number of earlier studies regarding effector memory and effector T cells, further validating these findings. Moreover, latent responsiveness of T cells after TCR stimulation may be an early marker of treatment response. Evidence is provided for the role of new markers such as CD38 for treatment resistance in melanoma, which might have direct therapeutic consequences. Activated CD4^+^ and CD8^+^ T cells showed a characteristic pattern in patients with irAE. Further studies with larger patient cohorts should be performed to further substantiate these findings. 

## 4. Materials and Methods

### 4.1. Study Design and Patients

Seventeen adult patients were enrolled in the exploratory study to identify putative markers. This was a non-interventional biomarkerstudy performed in one center. The study protocol was approved by the local ethics committee at the Medical Faculty at Leipzig University (reference number: 130/19-ek). The investigations were carried out following the rules of the Declaration of Helsinki of 1975, and patients were included after informed consent.

Patients were included with advanced unresectable stage IV melanoma, naïve for ICI therapies. Patients with progress after previous molecularly targeted treatment (such as BRAF inhibitors) or following adjuvant treatment with interferon alpha were allowed, as well as patients with adjuvant therapy with PD-1-inhibitors more than 6 months prior to study inclusion.

Patients were treated with ICI (pembrolizumab every three or six weeks, or nivolumab every two or four weeks, or a combination of nivolumab with ipilimumab every three weeks). Blood samples were collected at four pre-defined time points: (i) directly before (time point 1); (ii) directly after the first ICI infusion (time point 2); as well as (iii) directly before the second infusion (time point 3) after two or three weeks, depending on the ICI regimen; and (iv) after three months of treatment (time point 4). Additional sampling time points were determined for patients with either clinical evidence for irAEs or disease progression. Progression was radiologically assessed (response evaluation criteria in solid tumors, RECISTv1.1 criteria), and in case of suspicion of pseudoprogression, confirmed with repeated imaging at earliest time point after 4 weeks. 

Response was categorized as partial response (PR) or complete response (CR), whereas non-response included stable disease (SD) and progressive disease (PD) (RECIST criteria) after a minimum of six months. A maximum of five blood samples was taken from each patient. Follow-up of patients was after at least 6 months (maximum 18 months). 

### 4.2. Flow Cytometry

Peripheral blood samples from patients were collected to discriminate between specific cell populations and to assess: (i) a basic immune status, (ii) T cell differentiation, (iii) phosphorylation of signal transducer and activator of transcription 5 (pSTAT5), (v) PD-1- and CTLA4-expression on T cells, (vi) γδ-T cells, and (vii) human leukocyte antigen-HLADR isotype (HLA-DR) expression (Supplementary Tables S1 and S2). For (i), (ii), (vi), and (vii), patient’s peripheral blood was incubated with different antibody combinations as described by Boldt and co-workers (Supplementary Tables S1 and S2) (20, 21). For each sample, 100 µL of whole blood was incubated with an antibody cocktail specific for the desired cell populations [41,42]. After surface cell staining for 15 min at room temperature in the dark, erythrocytes were lysed by incubation with lysis buffer (BD Biosciences, Heidelberg, Germany) for 10 min. Following centrifugation and washing with PBS (Biochrom, Berlin, Germany), lymphocytes were fixed with 200 µL phosphate-buffered saline (PBS) containing 1% formaldehyde. The different populations consisted of T helper cells, cytotoxic T cells, B cells, NK cells, activated NK cells, NK T cells, activated CD4^+^T cells, activated CD8^+^ T cells, α/β T cells, γ/δ T cells, CD4/CD8 double-positive T cells, CD4/CD8 double-negative T cells, α/β CD4/CD8 double-negative T cells, γ/δ CD4/CD8 double-negative T cells, thymus migrant CD4+ T cells, thymus migrant CD8^+^ T cells, naive CD4^+^ T cells, naive CD8^+^ T cells, effector memory CD4^+^ T cells, effector memory CD8^+^ T cells, central memory CD4^+^ T cells, central memory CD8^+^ T cells, effector CD4^+^ T cells, effector CD8^+^ T cells, HLA-DR^+^ monocytes, regulatory T cells, PD-1^+^ T cells, PD-1^+^ T helper cells, cytotoxic T cells (for PD-1^+^ cells 24 h blank value or 24 h anti-CD3/-CD28 stimulated in vitro), CTLA-4^+^ T cells, CTLA-4^+^ T helper cells, CTLA-4^+^ cytotoxic T cells (for CTLA-4^+^ 24 h blank value or 24 h anti-CD3/-CD28 stimulated), and pSTAT5-expressing T cells (24 h blank value IL-2 stimulated or 24 h anti-CD3/-CD28 stimulated in vitro) (for more details see, Supplementary Table S2).

### 4.3. Analysis of STAT5-Phosphorylation—IL-2-Stimulation

200 µL EDTA-blood from patients was incubated with recombinant interleukin 2 (IL-2) (37 °C, 15 min). After that, whole blood was lysed by incubation with lysis/fixation buffer (BD Phosflow, BD Biosciences, Heidelberg, Germany) (12 min, 37 °C). After washing (500× *g*, 5 min) cells were permeabilized in Perm Buffer III (BD Biosciences) for 30 min on ice. After washing (500× *g*, 5 min) cells were stained with anti-pSTAT5 (pY705) (intracellular), anti-CD3 peridinin-chlorophyll proteins (PerCP)-Cy5.5, anti-CD4 phycoerythrin (PE), and anti-CD8 Alexa Fluor488 antibodies. Antibodies were purchased from BD Biosciences. After 1 h (at room temperature in the dark) cells were washed and fixed with 200 µL PBS containing 1% formaldehyde. An unstimulated panel was used as negative control.

### 4.4. Analysis of STAT5-Phosphorylation—CD3/CD28-Stimulation

PBMCs were isolated by density gradient centrifugation (1 × 10^6^ cells/ml RPMI, 10% fetal calf serum (FCS) + 1% penicillin/streptomycin), stimulated with anti-CD3/anti-CD28 antibodies (eBioscience, San Diego, CA, USA) at 37 °C, 5% CO_2_ for 24 h. The next day, cells were harvested, washed (500× *g*, 5 min) an incubated with lysis/fix buffer (BD Biosciences) for 12 min at 37 °C. After washing (500× *g*, 5 min) cells were stained as described above. An unstimulated panel was used as negative control.

### 4.5. Analysis of Lymphocyte Markers and PD-1-Expression

PBMCs were isolated by density gradient centrifugation as described above. PBMCs were then split into three parts, either left unstimulated, held under serum conditions for 24 h, or stimulated with anti-CD3/anti-CD28 for 24 h (at 37 °C). Cells were then stained by incubation with an antibody cocktail containing anti-CD8 PerCP, anti-CD4 V450, anti-CD3 V500, anti-CD45 APC-H7, anti-CD56 PE-Cy-7, anti-CD16 PE-Cy-7, and anti-PD1 PE for 15 min in the dark (Supplementary Table S1). Antibodies were purchased from BD Biosciences. In the next step, erythrocytes were lysed by incubation with lysis buffer (BD Biosciences) for 10 min. Following centrifugation and washing with PBS (Biochrom, Berlin, Germany), lymphocytes were fixed with 200 µL PBS containing 1% formaldehyde. 

### 4.6. Analysis of CTLA-4-Expression

PBMCs were isolated by density gradient centrifugation as described above. PBMCs were then split into three parts, either left unstimulated, held under serum conditions for 24 h, or stimulated with anti-CD3/anti-CD28 for 24 h (at 37 °C). Cells were then stained with anti-CD8 PerCP, anti-CD4 V450, anti-CD3 V500, anti-CD45 APC-H7, anti-CD56 PE-Cy-7, and anti-CD16 PE-Cy-7 for 15 min in the dark. After washing in PBS (500× *g*, 5 min) cells were permeabilized by FIX and Perm Buffer III (BD Biosciences) for 20 min. After washing, cells were incubated with anti-CTLA-4 APC (BD Biosciences) for 30 min in the dark. Cells were washed (2 x) and fixed with 200 µL PBS containing 1% formaldehyde.

### 4.7. Data Acquisition

For data acquisition, an eight color FACS Canto II flow cytometer (BD Biosciences) was used, equipped with a 405 nm violet laser, a 488 nm blue laser and a 647 nm red laser. For correct collection of fluorescent light, different band-pass filters and mirrors were used. For the violet laser: Horizon 450 channel (450/50) and Horizon 500 channel (510/50, 502LP); for the blue laser: SSC channel (488/10), FITC channel (530/30LP, 502LP), PE channel (585/42, 556LP), PerCP channel (670LP, 655LP), and PE-Cy7 channel (780/60, 735LP); for the red laser: APC channel (660/20) and APC-H7 channel (780/60, 735LP) were used. Data were analyzed using FACS DIVA (BD Biosciences) software. 

### 4.8. Clinical Data 

We collected demographic and clinical data such as age, sex, primary melanoma type, mutational status (BRAF; in BRAF wild type melanomas, additionally in individual cases NRAS and cKit, the latter especially for mucosal melanomas), tumor burden (including sum of diameters of measurable lesions and number of involved organs), levels of lactate dehydrogenase (LDH) as well as preceding melanoma therapies (e.g., adjuvant interferon alpha, targeted therapies). Furthermore, we obtained standard laboratory values including the tumor marker S100ß protein. 

### 4.9. Statistical Analysis 

Statistical analysis was performed using the statistic software R v4.0.2. Differences between responders and non-responders as well as patients with and without irAE were assessed by Mann–Whitney U test for each of the analyzed four time points. The Kruskal–Wallis test was applied for the analysis of time courses including *n* = 15 patients. Two patients, patients 12 and 14 (Table 1), were excluded from the final analyses because of an early change in treatment regimen to BRAF/MEK-inhibition and unknown cause of death, respectively. Differences between post-first dose levels (time point 2) and levels at the time point of first appearance of irAE were analyzed by Wilcoxon signed-rank test for all patients with observable irAE within the follow-up time. Based on the small sample size of the presented pilot study, the limited statistical power did not allow for statistical confirmation of the observed differences with proper adjustment for multiple testing. Instead, the most prominent differences were selected by a nominal (unadjusted) *p*-value of *p* < 0.05. For these features, the median as well as the median absolute deviation (MAD) of measured levels are presented for each group (R function MAD with parameter constant = 1). 

## Figures and Tables

**Table 1:** Clinical characteristics of melanoma patients included in this study.

Patient	Gender/Age ^a^	Melanoma Subtype/Site/Breslow in mm ^b^	BRAF/PD-L1 ^c^	ICI ^d^	Pre-Treatment ^e^	Tumor Burden in cm ^f^	Involved Sites ^g^	Response ^h^	irAE/Grade ^i^	S100/LDH ^j^
1	F/78	uNM/HN/1.8	WT/5%	4NIVO	RT	1.0	PUL	CR	none	-/+
2	M/76	uNM/T/1.4	WT/30%	1IPI/NIVO, 6NIVO	none	20.6	CER, LN, MUS, PER, SC	PR	irFAT/G3 irTHY/G2	-/-
3	F/70	NM/LE/3.9	WT/20%	4PEMBRO	aIFN	6.7	LN, PUL	PD	irMUC/G2	-/+
4	M/67	uALM/LE/3.1	WT/5%	4PEMBRO	none	3.4	HEP	PD	irHEP/G1	-/+
5	F/74	mucM/AN/12.0	WT/0% (cKIT WT)	1IPI/NIVO, 1NIVO	naRT	20.8	AN, HEP, PUL	PD	irHEP/G2	-/+
6	F/91	uNM/T/3.5	V600E/30%	4PEMBRO	none	3.9	CUT, HEP, PUL	PR	none	-/+
7	M/78	NM/T/9.6	WT/10%	2IPI/NIVO	none	15	HEP, LN, OSS	PR	irTHY/G2, irHYP/G2, irHEP/G1	+/+
8	M/75	uNM/UE/3.4	WT/0%	4PEMBRO	none	4.6	CUT, LN, SC	PR	none	+/-
9	F/41	NM/LE/3.1	V600E/0%	4IPI/NIVO, 1NIVO	aIFN, aNIVO, aTT	1.9	CER, LN	SD	none	+/-
10	F/57	SSM/T/0.8	V600E/5%	2IPI/NIVO, 4NIVO	TT	1.0	CER, PUL	PR	irHEP/G2	-/-
11	M/70	MNOS/T/1.4	WT/UKN	3IPI/NIVO, 1NIVO	none	13.6	CER, INT, PUL, LN	PR	irFAT/G2, irCOL/G2, irTHY/G1	+/+
12	M/77	uNM/T/9.0	V600E/5%	2NIVO	none	18.7	CUT, INT, LN, MUS, OSS, SPLE	n.a.	none	+/+
13	M/76	NM/HN/3.6	WT/UKN	1NIVO	none	16.9	ADR, BIL, OSS, PUL	PD	none	+/+
14	M/61	uSSM/OE/1.5	WTUKN	3PEMBRO	none	7.6	CUT, LN, PUL	n.a.	none	-/-
15	F/95	uNM/HN/11.0	WT/10%	5NIVO	none	7.5	ADR, HEP, MUS, PUL	PR	none	-/-
16	F/52	CUP/UKN/UKN	WT/2%	2IPI/NIVO	RT	2.6	PAN, MUS	CR	irDER/G1, irTHY/G1, irNEP/G1	-/-
17	M/48	CUP/UKN/UKN	V600E/0%	4IPI/NIVO	RT, TT	5.4	CER	PR	none	+/+

^a^ gender female (F) or male (M). ^b^ melanoma subtype (ulcerated (u), nodular melanoma (NM), superficial spreading melanoma (SSM), acral lentiginous melanoma (ALM), mucosal melanoma (mucM), melanoma not otherwise specified (MNOS)), melanoma of unknown primary (CUP), site of primary (head and neck (HN), upper extremities (UE), torso (T), lower extremities (LE)), anal (AN), unknown site (UKN)/Breslow thickness in mm. ^c^ BRAF mutation (V600) or wild type (WT); PD-L1 positivity in percent of melanoma cells in immune histochemical staining vs. staining unknown (UKN). ^d^ number of treatment cycles during immune monitoring and therapy regimen of immune checkpoint inhibitors: nivolumab monotherapy (NIVO; 240 mg Q2W or 480 mg Q4W),ipilimumab/nivolumab combination therapy (IPI/NIVO; IPI 3 mg/kg bodyweight and NIVO 1 mg/kg bodyweight), pembrolizumab monotherapy (PEMBRO; 200 mg Q3W or 400 mg Q6W). ^e^ pre-treatment: adjuvant treatment (a), neoadjuvant (na,) targeted therapy with BRAF/MEK-inhibitors (TT), ICI (NIVO, PEMBRO), radiotherapy (RT), interferon-α (IFN). ^f^ tumor burden in cm (target + non-target lesions). ^g^ in metastasis involved sites: adrenal (ADR), anal (AN), gallbladder/bile (BIL), cerebral (CER), cutaneous (CUT), hepatic (HEP), intestinal (INT), lymph node (LN), muscular (MUS), skeletal (OSS), pancreatic (PAN), peritoneal (PER), pulmonary (PUL), subcutaneous (SC), splenic (SPLE). ^h^ complete response (CR, all target lesions (TL) and non-target lesions (NTL) regressed), partial response (PR, sum of diameters TL + NTL at least −30%), progressive disease (PD, at least +20% in sum of diameters of TL + NTL), stable disease (SD, sum of diameters TL + NTL between −29% and +19%); response not available (n.a.) due to early treatment change to TT or unknown cause of death before response evaluation. ^i^ immune-related adverse event (irAE) during immune monitoring and grade according to CTCAE v5.0 (G1-5): irHepatitis (irHEP), irFatigue (irFAT; irFAT grade 1 was not assessed as irAE), irGamma-glutamyltransferasis elevated (irGGT), irThyroiditis (irTHY), ir lichenoid mucositis (irMUC), irHypophysitis (irHYP), irNephritis (irNEP). ^j^ S100 and LDH above upper level of normal (+) vs. normal values (-) at start of ICI.

## Data Availability

Data supporting results are provided as Supplementary Material, Table S4.

## Supplementary Materials

The following are available online at https://www.mdpi.com/article/10.3390/ijms22158017/s1.
