# Supplementary material for: Distinct Immune Signatures Indicative of Treatment Response and Immune-Related Adverse Events in Melanoma Patients under Immune Checkpoint Inhibitor Therapy
Source: Int J Mol Sci. 2021 Jul 27;22(15):8017. doi: 10.3390/ijms22158017 (PMC8348898; doi:10.3390/ijms22158017)
Supplement: Supplementary file 1 [file ijms-22-08017-s001.zip › 01_Supplementary Figures_summary_rev_20072021x.pdf]

Supplementary Figures

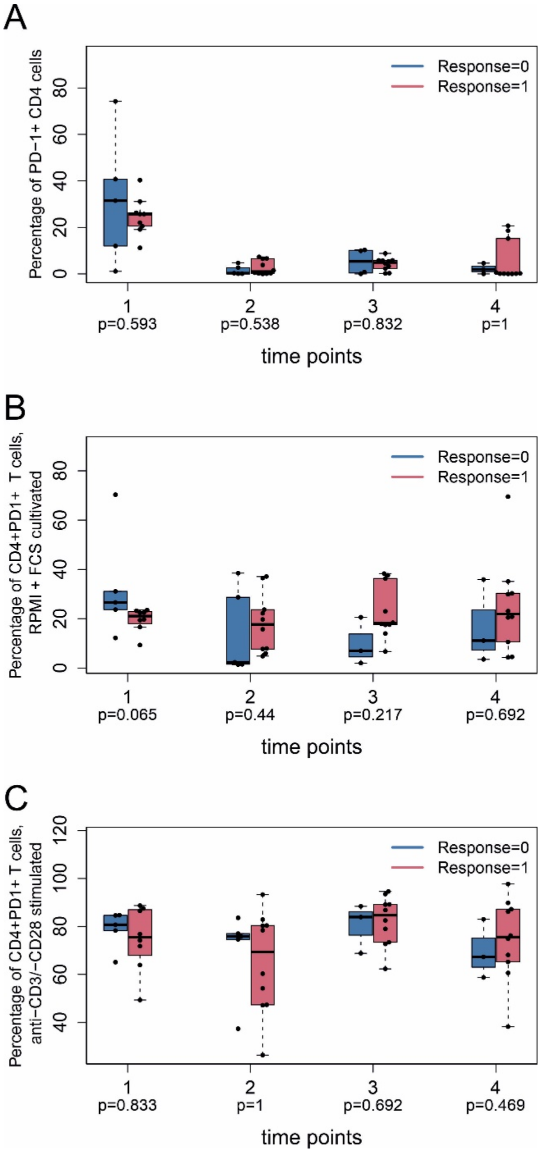

Figure S1: PD-1 expression on CD4^{+} T cells in ICI responders and non-responders. PBMC were taken from patients at indicated time points as described in Figure 1 and analyzed by flow cytometry (FACS). **(A)** PD1^{+}CD4^{+} expression without stimulation. **(B)** PD1^{+}CD4^{+} or PD1^{+}CD8^{+} expression in control cells after cell culture in 10% FCS. (C) PD1^{+} CD4^{+} or PD1^{+}CD8^{+} expression after anti-CD3/-CD28 stimulation. Nominal (un-adjusted) p-values (Mann-Whitney U test between responders and non-responders) are shown. Responders are indicated by response = 1, non-responders by response = 0. Data are shown as percentage of PD1^{+} cells of total CD4+ T cells.

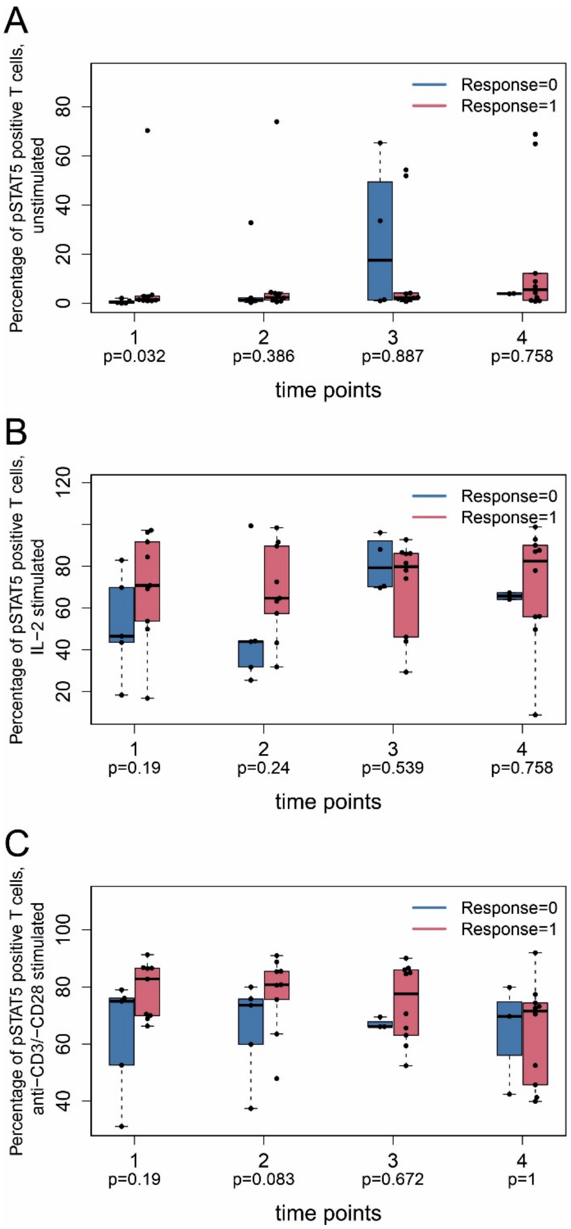

Figure S2. Intracellular expression of phosphorylated STAT5 (pSTAT5) in T cells in ICI responders and non-responders. PBMC were taken from patients at indicated time points as described in Figure 1 and analyzed by flow cytometry (FACS). **(A)** pSTAT5 expression without T cell stimulation. **(B)** pSTAT5 expression after T cell stimulation with IL-2 for 15 minutes. **(C)** pSTAT5 expression after T cell stimulation with antiCD3/-CD28. Nominal (un-adjusted) p-values (Mann-Whitney U test between responders and non-responders) are shown.

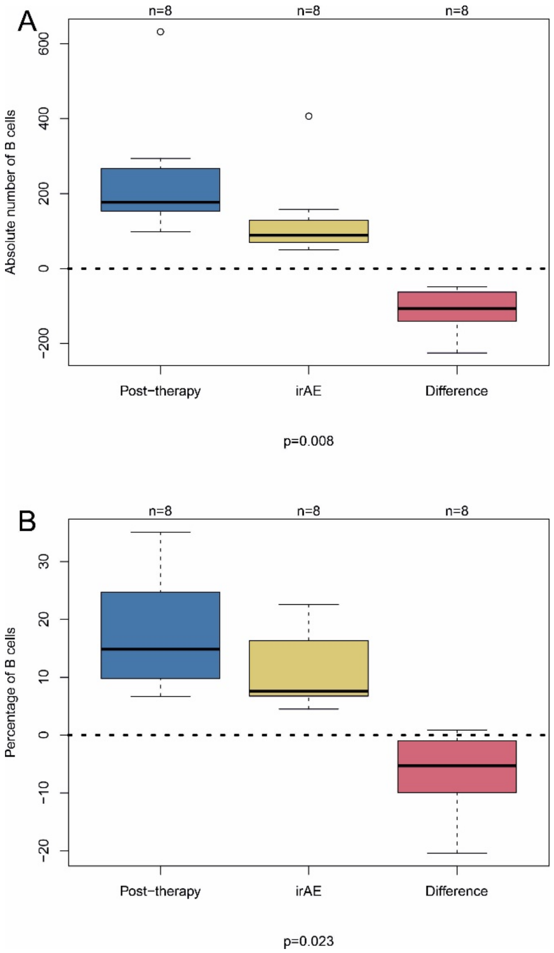

Figure S3. Absolute number and percentage of B cells in patients with irAE. PBMC were taken from patients immediately after first treatment on the same day (post-treatment; time point 2). In addition, PBMC were taken from patients at the time point of first appearance of adverse events (irAE) and analyzed by flow cytometry (FACS). The red boxplots illustrate the pairwise difference of post-therapy and irAE measurements. (A) Absolute number of B cells. (B) Percentage of B cells. Nominal (un-adjusted) p-values (Wilcoxon signed-rank test between post-therapy and irAE) are shown.
