# Supplementary material for: Distinct Immune Signatures Indicative of Treatment Response and Immune-Related Adverse Events in Melanoma Patients under Immune Checkpoint Inhibitor Therapy
Source: Int J Mol Sci. 2021 Jul 27;22(15):8017. doi: 10.3390/ijms22158017 (PMC8348898; doi:10.3390/ijms22158017)
Supplement: Supplementary file 1 [file ijms-22-08017-s001.zip › 01_Supplementary Table 1 antibodies_28102020x.pdf]

Supplementary Table S1: List of antibodies used in the present study.

Antibodies	Clone	Fluorochrome	Company	Catalogue number
**Immune status**				
CD3	UCHT1	V500	BD Horizon	561416
CD4	RPA-T4	V450	BD Horizon	560345
CD8	SK1	PerCP	BD	345774
CD16	B73.1	PE-Cy7	BD	335823
CD19	SJ25C1	APC	BD	345791
CD38	HB-7	PE	BD	345806
CD45	2D1	APC-H7	BD Pharmingen	560178
CD56	NCAM16.2	PE-Cy7	BD	335826
Anti-HLA-DR	L243	FITC	BD	347400
**α/β/γ/δ T-cells**				
Anti-TCR α/β	WT31	FITC	BD	333140
Anti-TCR γ/δ	B1	APC	BioLegend	331212
**T-cell differentiation**				
CD31	WM59	PE	BD Pharmingen	555446
CD45RA	HI100	FITC	BD Pharmingen	555488
CD45RO	UCHL1	PerCP-Cy5.5	BD Pharmingen	560607
CD197	3D12	Alexa Fluor 647	BD Pharmingen	557734
**Regulatory T cells**				
CD25	2A3	PE	BD	341011
CD39	TU66	FITC	BD Pharmingen	561444
CD127	HIL-7R-M21	Alexa Fluor 647	BD Pharmingen	558598
**Monocytes**				
CD14	M5E2	PE-Cy7	BD Pharmingen	557742
**CTLA-4/PD-1**				
CD152 (CTLA-4)	BNI3	APC	BD Pharmingen	555855
CD279 (PD-1)	EH12.1	PE	BD Pharmingen	560795
**Phosphorylated STAT 5**				
pStat5	47/Stat5(pY694)	Alexa Fluor 647	BD Phosflow	612599
