# Supplementary material for: Distinct Immune Signatures Indicative of Treatment Response and Immune-Related Adverse Events in Melanoma Patients under Immune Checkpoint Inhibitor Therapy
Source: Int J Mol Sci. 2021 Jul 27;22(15):8017. doi: 10.3390/ijms22158017 (PMC8348898; doi:10.3390/ijms22158017)
Supplement: Supplementary file 1 [file ijms-22-08017-s001.zip › 02_Supplementary Table 2 Panels_28102020x.pdf]

Supplementary Table S2: List of cell populations analyzed in the present study.

**Panel 1: Immune status**	
Leukocytes	
Lymphocytes	
T lymphocytes	$CD45^{+}CD3^{+}$
T-helper cells	$CD45^{+}CD3^{+}CD4^{+}CD8^{-}$
Cytotoxic T cells	$CD45^{+}CD3^{+}CD4^{-}CD8^{+}$
Ratio CD4/CD8	
B cells	$CD45^{+}CD19^{+}$
NK cells	$CD45^{+}CD3^{-}CD16/56^{+}$
Activated NK cells	$CD45^{+}CD3^{-}CD16/56^{+}$ $CD38^{+}$ $HLA\text{-}DR^{+}$
	$CD45^{+}CD3^{-}CD16/56^{+}$ $CD38^{+}$
	$CD45^{+}CD3^{-}CD16/56^{+}$ $HLA\text{-}DR^{+}$
NK T cells	$CD45^{+}CD3^{+}$ $CD16/56^{+}$
Activated $CD4^{+}$T cells	$CD45^{+}CD4^{+}CD8^{-}CD38^{+}HLA\text{-}DR^{+}$
	$CD45^{+}CD4^{+}CD8^{-}HLA\text{-}DR^{+}$
	$CD45^{+}CD4^{+}CD8^{-}CD38^{+}$
Activated $CD8^{+}$T cells	$CD45^{+}CD4^{-}CD8^{+}CD38^{+}HLA\text{-}DR^{+}$
	$CD45^{+}CD4^{-}CD8^{+}HLA\text{-}DR^{+}$
	$CD45^{+}CD4^{-}CD8^{+}CD38^{+}$
**Panel 2: α/β/γ/δ T cells**	
α/β T cells	$CD45^{+}CD3^{+}\alpha/\beta^{+}$
γ/δ T cells	$CD45^{+}CD3^{+}g/\delta^{+}$
Double-positive T cells	$CD45^{+}CD3^{+}CD4^{+}CD8^{+}$
Double-negative T cells	$CD45^{+}CD3^{+}CD4^{-}CD8^{-}$
α/β double-negative T cells	$CD45^{+}CD3^{+}CD4^{-}CD8^{-}\alpha^{+}/\beta^{+}$
γ/δ double-negative T cells	$CD45^{+}CD3^{+}CD4^{-}CD8^{-}\gamma^{+}/\delta^{+}$
**Panel 3: T cell differentiation**	
Thymus emigrant $CD4^{+}$	$CD45^{+}CD3^{+}CD4^{+}CD45RA^{+}CD31^{+}$
Thymus emigrant $CD8^{+}$	$CD45^{+}CD3^{+}CD8^{+}CD45RA^{+}CD31^{+}$
naive $CD4^{+}$	$CD45^{+}CD3^{+}CD4^{+}CD45RA^{+}CD45RO^{-}CCR7^{+}$
naive $CD8^{+}$	$CD45^{+}CD3^{+}CD8^{+}CD45RA^{+}CD45RO^{-}CCR7^{+}$
Effector memory $CD4^{+}$	$CD45^{+}CD3^{+}CD4^{+}CD45RA^{-}CD45RO^{+}CCR7^{-}$
Effector memory $CD8^{+}$	$CD45^{+}CD3^{+}CD8^{+}CD45RA^{-}CD45RO^{+}CCR7^{-}$
Central memory $CD4^{+}$	$CD45^{+}CD3^{+}CD4^{+}CD45RA^{-}CD45RO^{+}CCR7^{+}$

Central memory $CD8^{+}$	$CD45^{+}CD3^{+}CD8^{+}CD45RA^{-}CD45RO^{+}CCR7^{+}$
Effector $CD4^{+}$	$CD45^{+}CD3^{+}CD4^{+}CD45RA^{+}CD45RO^{-}CCR7^{-}$
Effector $CD8^{+}$	$CD45^{+}CD3^{+}CD8^{+}CD45RA^{+}CD45RO^{-}CCR7^{-}$
**Panel 4: HLA-DR on monocytes**	
HLA-DR on monocytes	$SSC^{+}CD14^{+}HLA\text{-}DR^{+}$
**Panel 5: Regulatory T cells**	
Treg	$CD45^{+}CD3^{+}CD4^{+}CD25^{+}$CD127low
IL2 receptor expression	$CD45^{+}CD3^{+}CD4^{+}CD25^{+}$
CD39	$CD45^{+}CD3^{+}CD4^{+}CD25^{+}$CD127lowCD39
**Panel 6: Detection of PD-1**	
On T lymphocytes	$CD45^{+}CD3^{+}PD1^{+}$
On T helper cells	$CD45^{+}CD3^{+}CD4^{+}CD8^{-}PD1^{+}$
On cytotoxic T cells	$CD45^{+}CD3^{+}CD4^{-}CD8^{+}PD1^{+}$
Blank value, 24 h blank value, 24 h anti^{-}CD3/$^{-}$CD28 stimulated	
**Panel 7: Detection of CTLA-4**	
In T lymphocytes	$CD45^{+}CD3^{+}CTLA4^{+}$
In T helper cells	$CD45^{+}CD3^{+}CD4^{+}CD8^{-}CTLA4^{+}$
In cytotoxic T cells	$CD45^{+}CD3^{+}CD4^{-}CD8^{+}CTLA4^{+}$
Blank value, 24 h blank value, 24 h anti-CD3/-CD28 stimulated	
**Panel 8: STAT5 phosphorylation**	
In T cells	$CD45^{+}CD3^{+}pSTAT5^{+}$
Blank value, IL-2 stimulated, 24 h blank value, 24 h anti-CD3/-CD28 stimulated	
