# Supplementary material for: Distinct Immune Signatures Indicative of Treatment Response and Immune-Related Adverse Events in Melanoma Patients under Immune Checkpoint Inhibitor Therapy
Source: Int J Mol Sci. 2021 Jul 27;22(15):8017. doi: 10.3390/ijms22158017 (PMC8348898; doi:10.3390/ijms22158017)
Supplement: Supplementary file 1 [file ijms-22-08017-s001.zip › 03_Supplement Table 3 Not Significant_25052021x.pdf]

Supplementary Table S3: List of cell populations that showed no significant differences before or during ICI treatment regarding the comparisons of treatment response and resistance and of immune-related adverse events with no immune-related adverse events.

**Not significant markers**	
Lymphocytes perc.	
T-lymphocytes abs. and perc.	$CD45^{+}CD3^{+}$
Double-positive T-cells perc.	$CD45^{+}CD3^{+}CD4^{+}CD8^{+}$
Double-negative T-cells perc.	$CD45^{+}CD3^{+}CD4^{-}CD8-$
NK cells abs. and perc.	$CD45^{+}CD3^{-}CD16/56^{+}$
NK T-cells perc.	$CD45^{+}CD3^{+}$ $CD16/56^{+}$
T-helper cells abs. and perc.	$CD45^{+}CD3^{+}CD4^{+}CD8^{-}$
Cytotoxic T-cells abs. and perc.	$CD45^{+}CD3^{+}CD4^{-}CD8^{+}$
Ratio CD4/CD8	
HLA-DR on activated NK cells perc	$CD45^{+}CD3^{+}$ $CD16/56^{+}$ $CD38^{+}HLA\text{-}DR^{+}$
HLA-DR on NK cells perc	$CD45^{+}CD3^{+}$ $CD16/56^{+}$ $HLA\text{-}DR^{+}$
Activated NK cells perc	$CD45^{+}CD3^{+}$ $CD16/56^{+}$ $CD38^{+}$
HLA-DR on cytotoxic T cells perc	$CD45^{+}CD3^{+}CD4\text{-}CD8^{+}HLA\text{-}DR^{+}$
Alpha/beta T-cells perc.	$CD45^{+}CD3^{+}a/b^{+}$
HLA-DR on monocytes perc.	$SSC^{+}CD14^{+}HLA\text{-}DR^{+}$
Tregs perc.	$CD45^{+}CD3^{+}CD4^{+}CD25^{+}CD127low$
CD25 on T cells	$CD45^{+}CD3^{+}CD4^{+}CD25^{+}$
$CD39^{+}$ Tregs perc.	$CD45^{+}CD3^{+}CD4^{+}CD25^{+}CD127lowCD39$
Thymus migrant $CD4^{+}$ abs. and perc.	$CD45^{+}CD3^{+}CD4^{+}CD45RA^{+}CD31^{+}$
Thymus migrant $CD8^{+}$ abs. and perc.	$CD45^{+}CD3^{+}CD8^{+}CD45RA^{+}CD31^{+}$
Naive $CD4^{+}$ abs. and perc.	$CD45^{+}CD3^{+}CD4^{+}CD45RA^{+}CD45RO^{-}CCR7^{+}$
Naive $CD8^{+}$ abs.	$CD45^{+}CD3^{+}CD8^{+}CD45RA^{+}CD45RO\text{-}CCR7^{+}$
Effector memory $CD4^{+}$ abs.	$CD45^{+}CD3^{+}CD4^{+}CD45RA^{-}CD45RO^{+}CCR7^{-}$
Effector memory $CD8^{+}$ abs.	$CD45^{+}CD3^{+}CD8^{+}CD45RA^{-}CD45RO^{+}CCR7^{-}$
Central memory $CD4^{+}$ abs. and perc.	$CD45^{+}CD3^{+}CD4^{+}CD45RA^{-}CD45RO^{+}CCR7^{+}$
Central memory $CD8^{+}$ abs. and perc.	$CD45^{+}CD3^{+}CD8^{+}CD45RA\text{-}CD45RO^{+}CCR7^{+}$
Effector $CD4^{+}$ abs.	$CD45^{+}CD3^{+}CD4^{+}CD45RA^{+}CD45RO^{-}CCR7^{-}$
Effector $CD8^{+}$ abs.	$CD45^{+}CD3^{+}CD8^{+}CD45RA^{+}CD45RO^{-}CCR7^{-}$
Extracellular PD1 on T-lymphocytes cells (blank value 0h)	$CD45^{+}CD3^{+}PD1^{+}$
PD1 on T-helper cells (blank value 0h, after 24h and 24h anti-CD3/-CD28 stimulated)	$CD45^{+}CD3^{+}CD4^{+}CD8^{-}PD1^{+}$
PD1 on cytotoxic T cells (24h anti-CD3/-CD28 stimulated)	$CD45^{+}CD3^{+}CD4^{+}CD8\text{-}PD1^{+}$
CTLA4 on T cells (blank value 0h, after 24 h and 24h anti-CD3/-CD28 stimulated)	$CD45^{+}CD3^{+}CTLA4^{+}$
CTLA4 in T helper cells (blank value 0 h, after 24h and 24h anti-CD3/-CD28 stimulated)	$CD45^{+}CD3^{+}CD4^{+}CTLA4^{+}$
CTLA4 in cytotoxic T-cells (blank value 0 h, after 24h and 24h anti-CD3/-CD28 stimulated)	$CD45^{+}CD3^{+}CD8^{+}CTLA4^{+}$
Perc. T cells pStat5A 15 min IL2 stimulated, 24 h anti-CD3/-CD28 stimulated	$CD45^{+}CD3^{+}STAT5^{+}$
Mean number of T cells pStat5A 24 h blank value, 24 h anti-CD3/-CD28 stimulated	$CD45^{+}CD3^{+}STAT5^{+}$

Perc., percentage; abs., absolute number
